# [Corrigendum] Downregulated caveolin‑1 expression serves a potential role in coronary artery spasm by inducing nitric oxide production *in vitro*

**DOI:** 10.3892/etm.2024.12535

**Published:** 2024-04-10

**Authors:** Xingmei Cao, Zhishuai Ye, Mingyu Jin, Shuai Yan, Xiantao Song, Rongchong Huang

Exp Ther Med 16:3567–3573, 2018; DOI: 10.3892/etm.2018.6646

Following the publication of the above paper, the authors have realized that the GAPDH western blot control bands featured in Fig. 2 on p. 3570 appeared incorrectly in the printed version of the article.

The authors have examined their original data, and have been able to both identify the correct blots, and realize how the error in the assembly of Fig. 2 occurred. The corrected version of Fig. 2 is shown opposite. Note that the error made in assembling this Figure did not affect the overall conclusions reported in the paper. All the authors agree with the publication of this corrigendum, and are thankful to the Editor of *Experimental and Therapeutic Medicine* for allowing them to publish this corrigendum. They also apologize to the readership for any inconvenience caused.

## Figures and Tables

**Figure 2:**
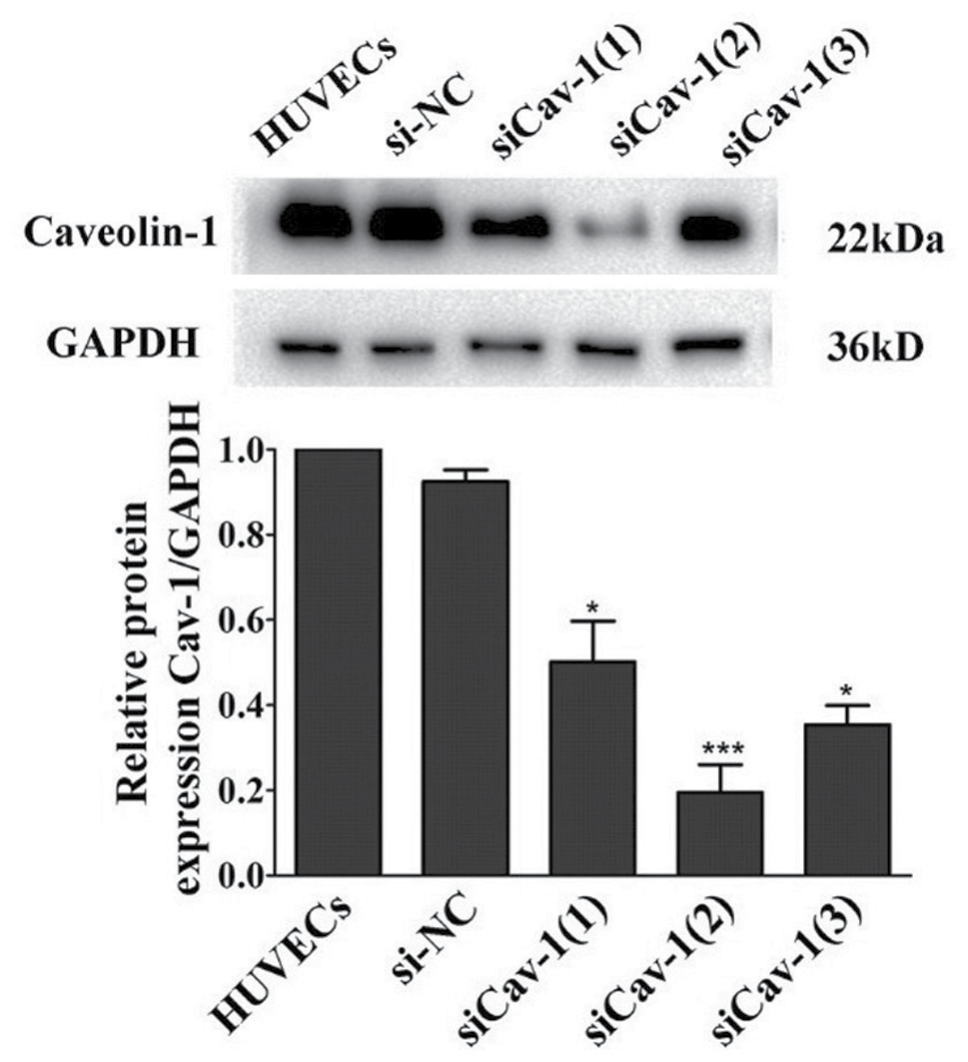
Western blot analysis of Cav-1 protein expression in HUVECs transfected with siCav-1(1), siCav-1(2) and siCav-1(3). GAPDH was used as a loading control. ImageLab software was used to quantify the immunoreactive band density, and GraphPad Prism version 5 software was used to generate the histogram. ^*^P<0.05 and ^***^P<0.001 vs. HUVEC. HUVECs, human umbilical vein endothelial cells; Cav-1, caveolin-1; GAPDH, glyceraldehyde 3-phosphate dehydrogenase; si, small interfering RNA; NC, negative control.

